# Carol Brayne: a life-course approach to prevent dementia

**DOI:** 10.2471/BLT.18.030318

**Published:** 2018-03-01

## Abstract

More research is needed to understand dementia and to find the approaches to dementia that are effective. Carol Brayne talks to Tatum Anderson.

Q: How did you become interested in the field of dementia?

A: In 1983, while working at the National Hospital for Neurology and Neurosurgery as a junior doctor, I knew I wanted to be a neuro-epidemiologist because I was interested in the causation of things and the kind of detective work that one does as an epidemiologist. For example, in such hospitals I noticed that many people with dementia were younger than we imagine people with dementia to be. When I went into this research field, very little was published on dementia and even less on the epidemiology of dementia. People had only just started to develop systematic approaches to measurement and investigation from a clinical perspective. A couple of years after I began researching older people, brain imaging was developed. Despite the advances in imaging techniques, the biggest challenge back then and still today is characterizing dementia, and separating it from other ways in which we age. When I started this research, there was no evidence base that we could use to determine the point at which normality changes into something that can be recognized as dementia syndrome.

Q: What kind of studies did you develop to find the answer?

A: There hadn’t been an in-depth study of any population, so I studied a particular age group of women in detail for the first time in the United Kingdom [of Great Britain and Northern Ireland]. What I pioneered then was bringing cutting-edge approaches to a population study, based on very detailed interviews that looked at a range of symptoms and a physical examination, including cardiovascular and neurological health, and blood tests. I designed the data collection to include all angles relevant to cognition and brain health, as known at the time. I interviewed 365 women aged 70–79 years in a small area in Cambridgeshire, and looked at their functional ability in daily activities, their mental function, and other variables associated with dementia. I also interviewed individuals who knew each of them well. As far as I know, this had not been done before in a population study.

Q: What did you discover?

A: In this cross-sectional study, we found that there was no clear division between the people who had dementia and those who did not, but that measures of dementia were continuously distributed across the population. This finding challenged the disease-based model at the time. In addition, this study and subsequent research showed that the prevalence of dementia doubles every five years in the over-65 age group. So, in the oldest age groups, you have a very high risk of dementia before you die. That has huge policy implications that have yet to be fully recognized.

“We can’t predict whether a person will or will not get dementia.”

Q: How did your research change the way dementia should be viewed?

A: People tend to talk about dementia as if it’s a disease that you have or you haven’t. But it’s a syndrome that impairs the mental function you had earlier in your life and that interferes with day-to-day activities. The response to dementia depends on the culture and society where you live. For example, if your country requires people to continue working to earn a living in the absence of social welfare, the loss of function will hamper your ability to remain independent. If you live in a society where elders are supported within households with multiple generations and few demands are placed on older individuals, decline due to dementia can be substantial before it is seen as a problem.

Q: You pioneered longitudinal studies on dementia. Why are they important?

A: To measure prevalence – the percentage of people at a given time with a condition – you need to do cross-sectional studies, but for incidence – which is the occurrence of new dementia – you must follow people at risk over long periods of time. Longitudinal studies also allow you to measure risk factors that occur before the onset of dementia, and you can use this to assess the risk later. Longitudinal studies take years to complete, but they are useful because they provide estimations of the number of people newly diagnosed with dementia per year, which is very important for health and social care planning services. These studies also show how important risk factors are.

Q: What are the risk factors and who is at risk?

A: Our longitudinal studies point to several big risk factors: diabetes, stroke, midlife obesity, midlife hypertension, depression, smoking, low education and low physical activity. Others have also been identified, such as hearing loss. Many of these factors are associated with socioeconomic inequality and are also key risk factors for noncommunicable diseases (NCDs). Dementia is now a topic of concern for the United Nations and the World Health Organization (WHO), along with the recognition of the key importance of NCDs globally. Social deprivation is likely to increase the risk of dementia and there are important protective factors. For example, we can’t predict whether a person will or will not get dementia, but we can say that people with a high level of education are at lower risk at any given age. Why? Because their brains are getting around problems that would otherwise cause difficulty.

Q: Dementia was predicted to explode as the older population of the United Kingdom increased. Tell us what your recent longitudinal studies showed?

A: People are changing. Vascular disease has dropped dramatically. For today’s population of older people, at least in the United Kingdom, we hypothesized that dementia would change as well, because risk factors for cardiovascular disease have changed. So, we repeated our study 20 years later, in 2010, and found that the estimated number of people with dementia had remained relatively stable despite a major increase in the proportion of the population in the oldest age groups.

Q: Why?

A: My hypothesis is that this population has lived through the period after the Second World War, when a socialized health and welfare system was introduced, reducing inequalities. Education was more widely available, child health improved, including immunization and access to health care, and people were better nourished. All of this resulted in people living longer in better health at old ages.

Q: How have your studies informed public health interventions in the United Kingdom?

A: Prevalence and incidence estimates as well as our estimates of care needs have all fed into policy development and long-term care planning. Since the mid-1990s the London School of Economics has been using these data for planning long-term care. Local authorities and National Health Service planners use our estimates to determine the proportion of the population general practitioners are likely to see with dementia and the scale of need related to disability. The change in dementia prevalence and incidence over time has helped shift the discussion towards what needs to be done earlier in life. As a result, Public Health England now has programmes encouraging population behaviour change related to known risks of dementia, faced by people from mid-life up to the age of 65 years. The Lancet Commission on Dementia and the Global Burden of Disease study have also drawn on our work.

Q: Other research suggests a dramatic increase in dementia across low- and middle-income countries by 2050. What might other countries learn from your research in the United Kingdom?

A: Where you have socioeconomic deprivation, you have a greater risk of developing dementia at an earlier age. Diagnosing dementia early has been a major focus in the United Kingdom and other countries. However, there is no empirical proof of effectiveness of such an approach, which is very similar to screening. Our work focuses on the need to address early factors that evidence suggests reduce our risk of dementia, and the need to provide decent services for those with dementia. Factors that seem likely to reduce the risk of developing dementia include: healthy parents, a birth without trauma, early life that allows the brain to develop to its fullest capacity, immunizations that prevent frequent sickness, access to education, a healthy diet, physical health, and social and intellectual engagement. The most effective way to tackle the global burden of dementia in ageing societies is by taking an approach that seeks to implement the evidence we have for each stage of the life course. This means enhancing protective factors in early, mid- and later life, appropriate availability of diagnostic approaches and holistic treatment for those people where dementia is affecting their lives and those around them and, finally, avoiding interventions that reduce the quality of life towards its end.

*Q: The Global Dementia Observatory is being developed by WHO to share best practices and evidence-based service planning in countries implementing the* Global action plan on public health response to dementia 2017–2025. *What kind of evidence should countries gather?*

A: Countries and localities need to carry out epidemiological needs assessments of the whole population for dementia risk at every age. We need to break out of the silos altogether – from policies on nutrition (better growth and health in early life) to road traffic accidents (reduce head injury)– they all need to take into account potential impact on healthy brains. We need to think about the health of the whole person within their social, environmental and cultural contexts and not just focus on a single thing, because dementia is far too complicated for that. Brain health could provide a very good indicator of how well a society functions for its individuals.

“Where you have socioeconomic deprivation, you have a greater risk of developing dementia at an earlier age.”

Q: What are the greatest challenges for your work?

A: Funding. We spend too much time trying to raise money for obvious research that is essential for our understanding of how dementia appears and affects different societies over time.

Q: What research gaps are there now?

A: We still know very little about dementia in the populations of low- and middle-income countries. Martin Prince has done a fantastic job with his studies, but there is nothing on the scale of the studies we have done here in the United Kingdom. We have been able to provide robust estimates of dementia for the whole country over several decades. Globally, most studies of dementia cohorts are not representative of populations and they are even less representative of the countries.

